# A Novel Urological Application of Hyaluronic Acid/Alginate Sheet to Prevent Adhesion Formation: A Technique Guide and Case Series

**DOI:** 10.7759/cureus.75849

**Published:** 2024-12-17

**Authors:** Dung Pham, Bryan Pham, Mitchell K Ng

**Affiliations:** 1 Urology, Children’s Memorial Hermann Hospital, Houston, USA; 2 Orthopaedic Surgery, Maimonides Medical Center, Brooklyn, USA

**Keywords:** chordee, hidden penis, hydrogel, orchiopexy, surgical adhesions, urology

## Abstract

Tissue gliding and mobility are paramount to the success of penile procedures. While postoperative healing is intended to protect, repair, and nourish injured tissues, an overzealous response often leaves painful and debilitating tethering between the corpora, including nerves, and surrounding tissues. This tethering interferes with the intended outcomes of surgery by preventing necessary gliding. An ideal implant that serves as a tissue gliding companion would be an easy-to-apply, temporary gliding surface that prevents unwanted tethering, bioresorbs completely, and flexibly addresses tissues that significantly contract postoperatively. Unfortunately, most commercially available options are difficult to use intra-operatively, are not designed to reduce friction, have variable efficacy, and have incomplete bioresorption. A promising new technology is a hydrogel made from alginate and hyaluronic acid (HA). Application of this hydrogel is revolutionizing orthopedic hand surgery, spine surgery, sports injury repair, and foot and ankle surgery with placement of gel/sheets around a range of soft tissue environments (joints, tendons, ligaments, peripheral nerves, vasculature) without issue. We present a case series of four patients undergoing various urologic procedures with the application of this novel bioresorbable hydrogel and their associated excellent clinical outcomes with no adverse events. We also discuss potential urologic indications for the use of this bioresorbable hydrogel along with technical intra-operative considerations.

## Introduction

Penile adhesions of the corporal bodies complicate common procedures such as circumcision, chordee, hidden penis, iatrogenic curvature of the corporal bodies, and orchiopexy [1]. For example, following circumcision penile adhesions are the most common complication with an estimated incidence of 15% [2]. Post-operative adhesions following penile procedures can often cause significant aesthetic and functional issues, leading to complications including pain, restricted movement, erectile dysfunction, and penile abnormalities [3]. The formation of adhesions, which occur when fibrous bands develop between tissues during healing, can lead to penile curvature, conditions resembling Peyronie’s disease, and shortening, which can profoundly impact sexual function and psychological health [4]. In addition, adhesions can involve nerves leading to reduced sensitivity and numbness. In more severe instances, adhesions can contribute to urethral strictures causing urinary difficulties, infections and occasionally requiring surgical intervention [4].

Poor patient outcomes associated with unwanted postoperative scarring are well-described throughout the literature, with decades of research resulting in products pouring onto the market to address the problems [5]. However, preventing the formation of these pathologic tissue bands tethering soft tissues that should remain separate is merely the first step in addressing the holistic problem. Unfortunately, products that demonstrate the ability to prevent adhesions, in turn, lead to secondary issues such as reduced healing, foreign body response, increased inflammatory response, and added bulk [5,6]. Animal- and human placental tissue-based products bioresorb via remodeling and replacement [6] leading to complications requiring surgical correction [7]. One prospective case series evaluating amniotic membrane allograft wrapped around zone 2 flexor tendon repairs was terminated early due to suboptimal results in 5 of 10 cases, specifically tendon rupture and continued marked stiffness [8]. In addition, collagen-based anti-adhesion implants may be difficult to resorb with one study demonstrating incomplete resorption even at six weeks post-op, potentially requiring revision to remove unnecessary bulk [9]. For penile procedures particularly, an ideal soft tissue protector product should not only protect against adhesions during critical healing and bioresorb completely alongside healing such that there is no trace of the implanted product, but also flexibly allow for postoperative tissue contraction [10].

A promising new product, VersaWrap (Alafair BioSciences, Inc., Austin, Texas) is a hydrogel that comprises hyaluronic acid (HA) and alginate, natural polysaccharides widely accepted not only for their anti-inflammatory and healing properties but also for seamless bioresorption profile [11]. HA maintains tissue hydration and supports cellular signaling essential for healing, while alginate provides structural integrity to the sheet, ensuring it remains in place and gradually degrades in synovial and mucosal-lined cavities without interfering with the natural healing processes [11]. VersaWrap is an ultrathin hydrogel sheet that transitions into a gelatinous, mucoadhesive layer, requiring no additional suturing to remain implanted. This gelatinous layer can be applied via a syringe, allowing unique application flexibility synergistic with penile procedures. VersaWrap's mechanism of action allows tissue gliding by immobilizing water onto its surface, thereby reducing tissue friction. VersaWrap is hydrophilic, but non-swelling, facilitating water diffusion such that small molecules, oxygen, growth factors, and nutrients may reach underlying tissues [12]. As a plant-based hydrogel, free of animal-derived or human tissue components, VersaWrap is non-immunogenic and has no concern for disease transmission [13]. VersaWrap is bioresorbed completely via hydrolysis and metabolic activity. The material is biocompatible and bioresorbable, degrading naturally over time, avoiding inflammatory or immune responses that could hinder recovery. By maintaining an environment conducive to tissue regeneration, VersaWrap is able to support healing while minimizing scar tissue formation.

Although being relatively novel, VersaWrap is used in a variety of surgical procedures to protect tendons [11] and peripheral nerves [10] from postoperative tethering. VersaWrap is touted for its flexible application, for being highly conformable to complex geometries, and for its transparent appearance allowing appropriate visualization of tissues during placement. VersaWrap is utilized across a range of soft tissue environments (joints, tendons, ligaments, vascular, peripheral nerves) without issue across a wide range of specialties, including but not limited to plastic surgery, hand surgery (digital nerve repairs, flexor tendon repairs, cubital tunnel release, microvascular cases and revision cases) [14], spine surgery (foraminotomy, laminectomy, discectomy) [13], and foot and ankle surgery (Achilles tendon repair, peroneal tendon reconstruction, ganglion cyst excision, tarsal tunnel release, ankle arthroscopy) [15]. Of particular interest is the use of VersaWrap in penile procedures to protect nerves and surrounding corporal tissues from postoperative tethering. In this paper, we present a case series of four patients undergoing various urologic procedures with the incorporation of this novel bioresorbable hydrogel, all of whom experienced excellent clinical outcomes with no associated adverse events.

## Case presentation

Case 1: Adult penile chordee

The patient is a 64-year-old male who presented to the clinic with a penile chordee. The patient had left-sided curvature since a young age, with progressive worsening over the past three years. The patient did not have pain with erections but had issues with left deflection of the urine and pain with intercourse. On the pre-operative exam, he had an uncircumcised phallus without any palpable plaques along the penile shaft. The patient brought photographs demonstrating a 45- to 50-degree left-sided curvature during an erection. After a discussion of surgical options, the patient elected for penile plication due to a lower risk of post-operative erectile dysfunction but with a potential risk for shortening of the penis.

**Figure 1:**
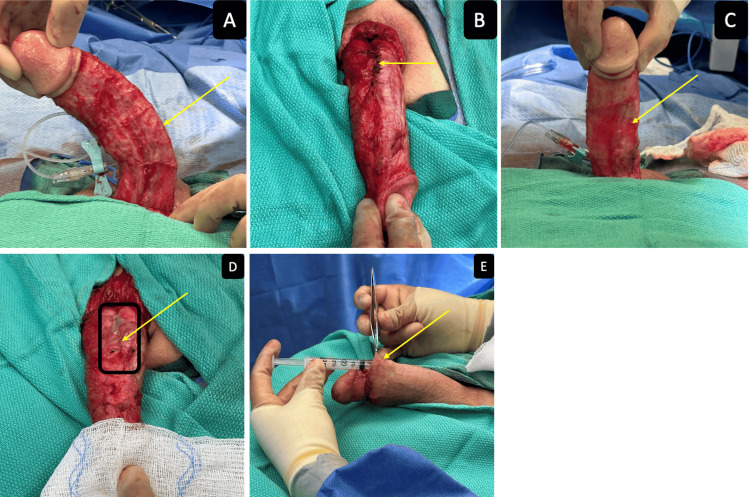
(A) Left-sided 45-degree curvature; (B) Penile plication with 2-0 prolene; (C) Resolution of curvature; (D) VersaWrap sheet over plication (as outlined by black square); (E) Demonstration of VersaWrap gel applied under dartos fascia and penile skin.

Intra-operatively, the penis was degloved and an artificial erection was performed revealing a 45-degree left-sided curvature (Figure 1A). To decrease the risk of penile shortening, the penis was pulled out and a 2-0 Prolene (Ethicon, Inc., Somerville, USA) was used to anchor Buck’s fascia to deep dermal tissue at the 3 and 9 o’clock position to the corresponding Buck’s fascia on each side. A 2-0 Prolene was also used to anchor the fascia of the scrotum to the corresponding Buck’s fascia at the 5 and 7 o’clock position with an interrupted suture on each side. The patient underwent plication with three 2-0 Prolene sutures (Figure 1B), with repeat artificial erection demonstrating subsequent resolution of penile curvature (Figure 1C). VersaWrap was placed as a sheet over penile plication sutures and then was applied as a gel under dartos fascia and penile skin immediately prior to closure (Figures 1D-1E).

VersaWrap gel was manipulated to ensure coverage around the penile shaft and on the urethra to prevent unwanted tissue tethering. At a six-week follow-up, the patient had no loss of penile length, straight phallus during erections, no tissue tethering, no keloid formation, no reported pain, and reported the ability to resume intercourse.

Repair of the penile curvature is best achieved by minimizing the chances of postoperative scarring. Postoperative scarring of the overlying tissue causes unwanted tethering and results in poor cosmetic and functional outcomes. Postoperative scarring can also lead to increased pain from erections during the healing period as well as after the healing period. VersaWrap placed onto the penile plication sutures, under the dartos fascia and the penile skin prevents unwanted tissue tethering to improve postoperative pain, cosmesis, functional outcomes, and patient satisfaction.

Case 2: Pediatric inguinal orchiopexy

The patient is a three-month-old male presenting to the clinic with bilateral cryptorchidism, born healthy from a full-term uncomplicated pregnancy. There was no significant or known family history of cryptorchidism with low concern for genetic disorders. The patient had problems with a deflected urinary stream leftwards. On exam, the patient had an uncircumcised phallus with 100-degree clockwise penile torsion and ventral curvature of 30 degrees. The right testicle was not palpable, while the left was palpated near the left inguinal canal. The patient’s parents elected for diagnostic laparoscopy, possible first-stage orchidopexy, inguinal hernia repair, left inguinal orchidopexy, repair of penile curvature, and penile torsion.

**Figure 2:**
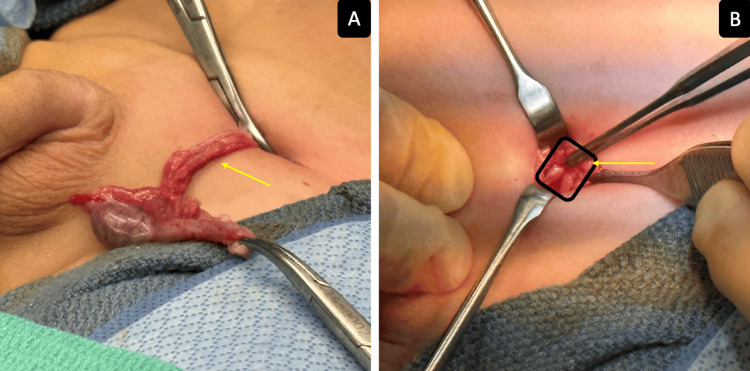
(A) Left inguinal orchiopexy; (B) VersaWrap placed over the spermatic cord and nerves.

Intra-operatively, diagnostic laparoscopy revealed a right intra-abdominal testicle appearing normal, while the left inguinal canal revealed a hernia with a normal vas deferens and spermatic vessels exiting the inguinal canal. A left inguinal incision was made, identifying the spermatic cord and hernia sac. The spermatic cord, the testicle, and the hernia were dissected up to the left internal inguinal ring (Figure 2A). The cremasteric muscle was split and divided. The vas deferens and the spermatic vessels were dissected off the hernia sac. The hernia sac was ligated. A dartos pouch was then created, and the testicle was brought down and anchored to the scrotum using a 4-0 Prolene interrupted suture. A VersaWrap sheet was placed over the spermatic cord, vas deferens, and ilioinguinal nerve (Figure 2B).

A first stage Fowler-Stephens orchiopexy was performed on the right testicle. The penis was degloved and an artificial erection revealed a 30-degree ventral curvature. Penile plication was performed with a 3-0 Prolene suture. The penile torsion was corrected using a dartos flap. The VersaWrap was placed around the corpora, urethra, the plication site, underneath the penile skin, the dartos layer, and peripheral nerves. VersaWrap was applied around the spermatic cord to prevent tethering, so the testicle stayed in the dependent scrotum, with gliding of the spermatic cord potentially reducing postoperative pain and preventing tethering of the cord, which could lead to iatrogenic retraction of the testicle that can result in a re-do orchidopexy. At a four-week follow-up, the patient did excellent requiring minimal pain medication and the left testicle remained in the dependent scrotal position and was very mobile. The phallus remained straight with minimal swelling and a straight urinary stream. He returned to normal activity without restriction. The patient was brought back six months later for a laparoscopic second-stage orchidopexy. The gubernaculum was ligated using the LigaSure device (Medtronic plc, Dublin, Ireland). Sharp dissection was used to free the testicle, vas deferens, and vasal vessels from the peritoneum. A dartos pouch was then created, and neo-canal was created lateral to the bladder. A Maryland grasper was then used to go through the neo-canal and into the scrotum. A trocar sheath was then grabbed by the Maryland grasper and pulled into the abdomen. The trocar was then inserted into the sheath. Graspers were used to bring the testicle out through the trocar. The trocar was removed, and the testicle was anchored using a 4-0 Prolene. VersaWrap gel was applied to the neo-canal using the laparoscopic applicator. The gel was deposited into the neo-canal, around the intra-abdominal vas deferens, and the dartos muscle in the scrotum. The patient was seen back in six weeks, three months, and one year. The right testicle felt normal, and in the scrotum, the vas deferens were mobile, and similar in size based on ultrasound and physical exam.

Retraction of the testicle after orchiopexy is a known risk and certain surgical maneuvers are made to try to mitigate the risk, such as narrowing the neck of the dartos pouch. Postoperative inflammation can cause scarring and if the testicle retracts during the postoperative period, the scarring can cause the testicle to remain in an ectopic location. VersaWrap is applied around the spermatic cord and nerve bundle to prevent tethering, so the testicle stays in the dependent scrotum. The gliding of the spermatic cord and nerve bundle reduces postoperative pain as well. For the second stage, the VersaWrap is applied to the vas deferens, vasal vessels, and neo-canal. A re-do of second-stage orchidopexy is a very daunting task due to significant adhesions and the high risk of blood supply disruption.

Case 3: Adult Peyronie’s disease

The patient is a 48-year-old male with atraumatic left-sided penile curvature that has been ongoing for over a year. He initially had pain with erections with no reported pain over the past six months preceding operative intervention. The patient had a 90-degree dorsal curvature, with a 3x2 centimeter (cm) plaque near the base of the phallus. The plaque has been stable for about a year. He has pain with penetration. The patient elected to undergo excision of the plaque and grafting using Tutoplast (Zimmer Biomet, Warsaw, USA).

**Figure 3:**
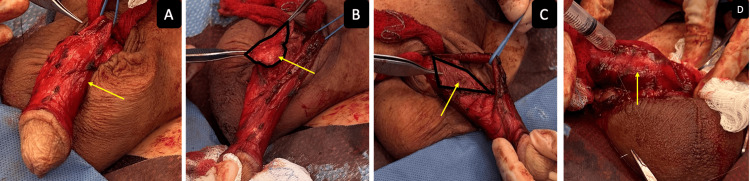
(A) Neurovascular bundle dissected (identified by silver forceps); (B) Penile plaque excised (as highlighted by black line); (C) Corpora cavernosa defect (as highlighted by black line); (D) VersaWrap placed over penile graft (contained with syringe).

Intra-operatively, the penis was degloved and the neurovascular bundle was released from the corpora cavernosa and penile plaque (Figure 3A). The penile plaque was outlined and excised (Figures 3B-3C). The defect was closed with Tutoplast, with VersaWrap applied over the graft and around the neurovascular bundle (Figure 3D).

VersaWrap sheet application was performed around the corpora, urethra, dartos layer, underside of the penile skin, and the neurovascular bundle to protect nerves and potentially stimulate nerve repair, thereby minimizing post-operative pain and preventing overlying tissue from adhering to surrounding tissues. At the three-week follow-up, the patient did very well with no reported pain, minimal penile swelling, and intact penile sensation, and was able to achieve spontaneous erections without pain.

Peyronie’s disease can cause significant discomfort and risk of penile injury during intercourse. Correction of severe penile curvature associated with large penile plaques often requires invasive surgery to excise the plaque followed by grafting to close the defect, which can potentially cause erectile dysfunction or result in nerve injury. VersaWrap placed around the neurovascular bundle helps protect the nerves and potentially stimulate nerve repair. Application of VersaWrap over the graft site, corpora, urethra, penile skin, and dartos layer helps minimize pain postoperatively by preventing overlying tissue from tethering and pulling on the delicate closure, especially during spontaneous erections.

Case 4: Pediatric hidden penis

**Figure 4:**
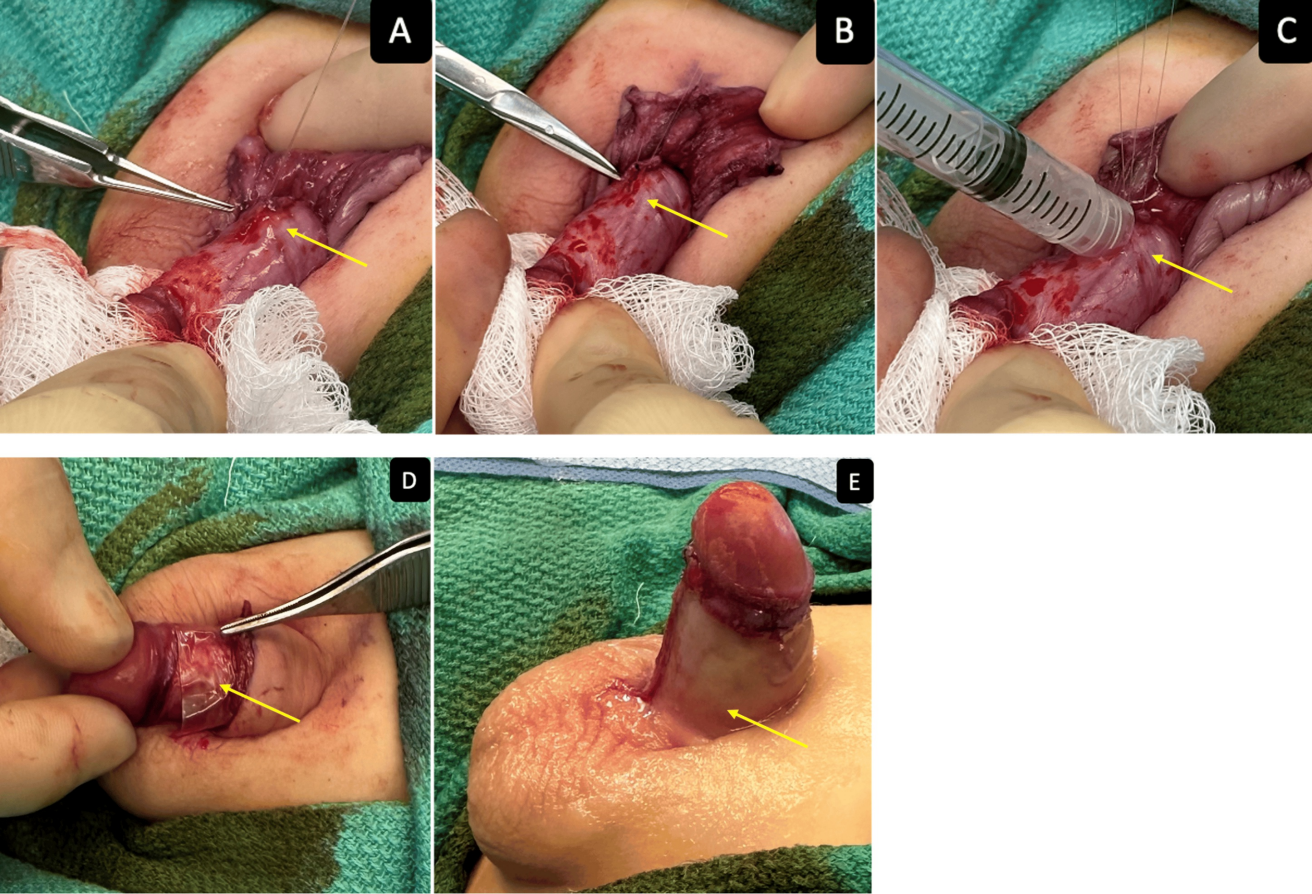
(A) Penile plication sutures (highlighted by forceps); (B) Additional penile plication sutures; (C) Hyaluronic acid (HA)/alginate gel placed over corpora and nerves and on penile anchor sutures (contained within syringe); (D) VersaWrap sheet placed over penile plication sutures (highlighted by forceps); (E) Resolution of chordee.

The patient is an 18-month-old male who presented to the clinic with a hidden penis. The patient was born at full term from an uncomplicated pregnancy and was otherwise healthy. His parents were concerned due to the hidden appearance of the phallus along with the curvature of the phallus. On physical exam, there was a hidden penis with penoscrotal webbing and ventral curvature of the phallus. The parents elected for chordee repair, correction of the hidden penis, and the penoscrotal webbing (Figure 4).

VersaWrap was placed around the corpora, urethra, plication site, underneath the penile skin, dartos layer, and underneath the scrotal skin to prevent tethering of the penile and scrotal skin to the underlying structure. By allowing the tissue gliding, this approach helps thereby decreasing poor cosmetic outcomes, post-operative pain from spontaneous erections, and the need for revision surgery. The patient did very well at the four-week follow-up, requiring minimal pain medications and returning to normal activity, with a straight phallus and well-healed surgical incision.

Hidden penis can be cosmetically and functionally debilitating. Penile anchoring sutures are used to correct the condition by further freeing up the corpora and urethra from the surrounding tissue and then re-anchoring the penile corpora in four quadrants. Tethering of the penile skin and dartos fascia to the corpora can cause poor results due to the adhesions pulling the corpora bodies back into the pelvis and dimpling of the skin. To prevent tethering of the dartos fascia and penile skin, VersaWrap is placed around the corpora, nerves, urethra, dartos fascia, penile skin, and scrotal skin. By allowing gliding, VersaWrap also reduces postoperative pain from spontaneous erections.

## Discussion

This paper demonstrates the use of a novel HA/alginate implant, VersaWrap, in various urological cases (penile plication, orchidopexy, penile plaque grafting, and chordee repair) to prevent unwanted postoperative tethering. Our case series demonstrates no significant adverse events with excellent clinical outcomes at post-operative follow-up (Table 1).

**Table 1:** Summary of urological cases where VersaWrap was utilized with associated clinical outcomes. HA: hyaluronic acid; HPI: history of present illness

Patient HPI	Diagnosis	Procedure performed	VersaWrap application	Outcome
A 64-year-old male with worsening left penile curvature	Penile Chordee	Penile plication	HA/alginate application over plication sutures, injected under dartos fascia, penile skin, corporal bodies, and urethra	Prevent unwanted tethering to improve post-op pain, cosmesis, and functional outcomes. A six-week follow-up no loss of length, phallus straight during erections, no pain, and able to resume intercourse.
A three-month-old male with bilateral cryptorchidism, uncomplicated birth	Pediatric inguinal orchiopexy	Diagnostic laparoscopy, bilateral orchidopexy with repair of penile curvature/torsion	HA/alginate applied around corpora and spermatic cord covering plication sutures, penile skin, corporal bodies, and dartos fascia	Prevent tethering of the spermatic cord to the surrounding structure so the testicle stays in the dependent scrotum, reducing postoperative pain and iatrogenic retraction of the testicle. A four-week follow-up patient required minimal pain medication, the testicle was in dependent scrotum position, and the spermatic cord was very mobile, with minimal swelling
A 48-year-old male with 90-degree left-sided penile curvature, pain with erections	Adult Peyronie’s	Incision and grafting of penile plaque	HA/alginate applied over graft, neurovascular bundle, penile skin, corporal bodies, urethra, and dartos layer	HA/alginate is placed around the neurovascular bundle protecting the nerve, stimulating nerve repair, and minimizing pain. At the six-week follow-up patient with no pain with minimal swelling, and intact penile sensation was able to achieve pain-free erections with good cosmesis and no tethering of the skin to the corpora.
An 18-month-old male presenting with a hidden penis, born full term from uncomplicated pregnancy	Pediatric hidden penis	Chordee repair, correction of hidden penis	HA/alginate was applied over the penile anchor and plication sutures, penile skin, corporal bodies, urethra, and dartos fascia	Placed around plication sutures/corpora to prevent tethering of dartos fascia, placed around anchor sutures to prevent tethering of the anchor sites to the penile skin. At the four-week follow-up patient with minimal pain medications, back to normal activity with well-exposed phallus.

There is growing literature that the use of VersaWrap is safe and effective, with no adverse events or revision surgery reported [12-15]. Ultimately, the purpose of its use is to allow tissue gliding and decrease post-operative tethering in a local soft tissue environment, thereby improving pain and function, and reducing revisions [11].

The integration of VersaWrap (HA/alginate) into routine urological procedures, as practiced at the senior author's institution, reflects the growing recognition of the material’s versatility and ease of use. Its application is not limited to a single type of surgery but spans various urological interventions, particularly those that involve delicate soft tissues prone to adhesion formation. The flexibility of applying VersaWrap as a sheet or as a gel, without the need for sutures or additional fixation devices, enhances its practical utility in the operating room [11,16]. Moreover, the ability to combine the sheet with its gel form in more complex revision cases demonstrates the flexibility of the material in addressing different anatomical challenges [11].

The decision to routinely apply VersaWrap in surgeries such as penile plication, orchidopexy, and chordee repair highlights its potential in preventing postoperative complications related to adhesion formation. As the senior author’s experience suggests, this material has become integral to protecting sensitive structures, such as the nerves, preventing loss of sensation, and adhesions of the penile and/or scrotal skin to the underlying penile nerves, corporal bodies, and urethra. This is accomplished by facilitating smooth tissue gliding during the healing process. This protection is particularly important in surgeries where preserving the function and mobility of these structures is vital for both patient outcomes and quality of life.

One of the most promising applications of VersaWrap is in revision surgeries, where the risk of adhesion formation is significantly higher due to previous surgical scarring and fibrosis [17,18]. In such cases, postoperative tethering can lead to further complications, such as limited tissue mobility, increased pain, and functional impairment. The ability to use both the sheet and gel form of VersaWrap in these scenarios provides surgeons with an additional tool to minimize these risks. As demonstrated in our case series, the intra-operative application is intuitive and does not add significant time to the procedure, making VersaWrap an attractive option in revision surgeries where preventing recurrence is a priority. Without its application, post-operative complications related to adhesion formation including pain, dysfunction, and restricted tissue movement would be at risk of higher prevalence.

While adhesions are a near-universal occurrence in surgical procedures, the use of this novel anti-adhesion barrier could represent a paradigm shift in how surgeons approach both primary and revision surgeries. By reducing the likelihood of tissue tethering and recurrent adhesions, VersaWrap may not only improve immediate postoperative outcomes but also reduce the need for future surgical interventions [12,13]. To this end, the primary surgeon of this study is now routinely using VersaWrap during almost all urologic procedures, particularly pediatric ones or revision cases with a higher risk of adhesion/scar formation.

Although this paper focuses on the application of VersaWrap in Urology, the material has shown promise across various surgical subspecialties. Its use in tendon, ligament, vessel, and nerve surgeries in the setting of multiple soft tissue environments underscores the broad potential of this bioresorbable hydrogel to lead to improved patient outcomes [13-15,18]. This cross-disciplinary utility suggests that VersaWrap could become a staple in surgical practice beyond urology, with applications in orthopedics, plastic surgery, neurosurgery, general surgery, and beyond [12-15]. Within urology specifically, the ability to apply this material in cases ranging from penile plaque grafting to orchidopexy further exemplifies its adaptability. Given the success seen in these cases, it is conceivable that future studies could investigate its use in other complex urological surgeries, such as cystectomy, bladder augmentation, or complex urethral reconstructions, where postoperative adhesions could compromise functional outcomes.

While the results of this case series are promising, the relatively small number of patients limits the generalizability of the findings. Larger studies (e.g. randomized clinical trials), with more diverse patient populations and longer follow-up periods, are necessary to fully understand the long-term effects of VersaWrap on adhesion prevention and surgical outcomes. Furthermore, while no adverse events have been reported in this series or in literature to date, continued surveillance is important to ensure the material remains safe and effective across different patient demographics and procedural complexities. Another area of future research could involve exploring the optimal timing for applying VersaWrap, as well as assessing how the material interacts with different types of tissue during healing. Investigating the material's performance in high-risk patients, such as those with a history of poor wound healing or those undergoing multiple surgeries, could also provide valuable insights.

## Conclusions

There is a high incidence of post-operative adhesions after urological surgery. This paper highlights the use of a novel HA acid/alginate sheet in various urological procedures to prevent adhesion formation, including penile plication, orchidopexy, penile plaque grafting, penile torsion repair, correction of penoscrotal webbing, and chordee repair. The primary goal of using this material is to reduce postoperative adhesions in soft tissue, thereby minimizing tethering, improving pain and function, and reducing recurrence rates. Our case series showed no significant adverse events and excellent clinical outcomes at postoperative follow-up. The growing body of literature supports the safety and efficacy of VersaWrap, with no adverse events or need for revision surgery reported. As the literature supporting the safety and efficacy of VersaWrap continues to grow, its use may become more widespread across multiple surgical specialties. Ultimately, this technology holds the potential to improve postoperative recovery, reduce pain and complications, and enhance long-term functional outcomes in both primary and revision surgeries. Future studies should be directed towards more fully characterizing the beneficial effects of VersaWrap and associated long-term patient outcomes.
